# Investigating the Adoption of Clinical Genomics in Australia. An Implementation Science Case Study

**DOI:** 10.3390/genes12020317

**Published:** 2021-02-23

**Authors:** Stephanie Best, Janet C. Long, Clara Gaff, Jeffrey Braithwaite, Natalie Taylor

**Affiliations:** 1 Australian Institute of Health Innovation, Macquarie University, Sydney, NSW 2113, Australia; janet.long@mq.edu.au (J.C.L.); jeffrey.braithwaite@mq.edu.au (J.B.); 2 Australian Genomics Health Alliance, Murdoch Childrens Research Institute, Melbourne, VIC 3052, Australia; 3 Melbourne Genomics Health Alliance, Walter and Eliza Hall Institute, Melbourne, VIC 3052, Australia; clara.gaff@melbournegenomics.org.au; 4 Department of Paediatrics, University of Melbourne, Melbourne, VIC 3010, Australia; 5 Cancer Research Division, Cancer Council New South Wales, Sydney, NSW 2011, Australia; natalie.taylor@nswcc.org.au; 6 Faculty of Medicine and Health, University of Sydney, Sydney, NSW 2050, Australia

**Keywords:** clinical genomics, implementation science, Theoretical Domains Framework, COM.B

## Abstract

Despite the overwhelming interest in clinical genomics, uptake has been slow. Implementation science offers a systematic approach to reveal pathways to adoption and a theory informed approach to addressing barriers presented. Using case study methodology, we undertook 16 in-depth interviews with nongenetic medical specialists to identify barriers and enablers to the uptake of clinical genomics. Data collection and analysis was guided by two evidence-based behaviour change models: the Theoretical Domains Framework (TDF), and the Capability, Opportunity Motivation Behaviour model (COM-B). Our findings revealed the use of implementation science not only provided a theoretical structure to frame the study but also facilitated uncovering of traditionally difficult to access responses from participants, e.g., “safety in feeling vulnerable” (TDF code *emotion*/COM-B code *motivation*). The most challenging phase for participants was ensuring appropriate patients were offered genomic testing. There were several consistent TDF codes: *professional identity, social influences*, and *environmental context and resources* and COM-B codes *opportunity* and *motivation*, with others varying along the patient journey. We conclude that implementation science methods can maximise the value created by the exploration of factors affecting the uptake of clinical genomics to ensure future interventions are designed to meet the needs of novice nongenetic medical specialists.

## 1. Introduction

The adoption of clinical genomics in Australia is at a tipping point. National and state-based investment and research have created evidence of the value of genomics in a wide range of clinical specialities [1] and public funding is supporting the use of genomic testing for a number of clinical indications e.g., intellectual disability or global developmental delay in Australia [2]). Yet take up of genomics remains slow [3]. The use of implementation science approaches can help reveal influences on adoption and identify mechanisms to support uptake. 

However, criticism can be mobilised towards the field of health care implementation and improvement due to the plethora of theories, frameworks and models available to the researcher [4]. Offering such a bewildering array of approaches can paralyse even the most enthusiastic healthcare improvement, implementation or research leads, leading to a retreat away from theory informed methods and a reliance on intuition and personal knowledge. Various authors have considered how to make the use of theory in quality improvement more accessible (e.g., [5]) and ensure implementation science is more user friendly (e.g., [6]). These approaches can help promote the idea of “implementation literacy”, encouraging a common understanding and language to ensure evidence gets into practice. We build on these approaches to gather critical insights in the take up of clinical genomics and consider opportunities for implementation science to be employed while studying complex interventions.

As the uptake of clinical genomics matures beyond the realm of clinical geneticists, engaging nongenetic medical specialists will be vital to ensure service provision which optimises sustained patient access. Currently, many nongenetic medical specialists would prefer to refer to clinical genetics for genetic/genomic clinical decision making and we know that uptake of genomic testing by physicians is influenced by their knowledge, confidence in how to use genomic results and beliefs about genomic medicine [7,8]. To gather a more in-depth understanding of physicians’ perceptions, we used case study methodology to examine the behavioural drivers and experiences of early, nongenetic medical specialist adopters who were applying genomics in clinical healthcare settings. Case study methodology allows researchers to examine complex phenomena within “real life” settings [9,10,11,12]. The case study approach encourages consideration of multiple strategies, methods or theories to capture the complexity of the “case” in hand [13], allowing us to examine the “what”, “how” and “why” something happens [14]. This freedom allowed us to generate two aims for this study, (i) to establish priority areas to focus theory-informed implementation efforts to promote the future adoption of clinical genomics for novice nongenetic medical specialists and (ii) to critically examine the utility of implementation science methods when exploring factors affecting the uptake of evidence into practice.

## 2. Materials and Methods

### 2.1. Context

Amidst the global shift towards the adoption of genomic sequencing in clinical practice [1], Australia has employed national and regional strategies to promote uptake. Both the national collaborative research programme, Australian Genomics Health Alliance [15] and state-based programs, e.g., Melbourne Genomics Health Alliance [16] have identified the importance of developing “real world” evidence for the implementation of genomics in clinical practice. As a result, implementation research has been undertaken alongside these clinical trials in the form of hybrid effectiveness/implementation designs [17].

### 2.2. Research Design

**Figure 1:**
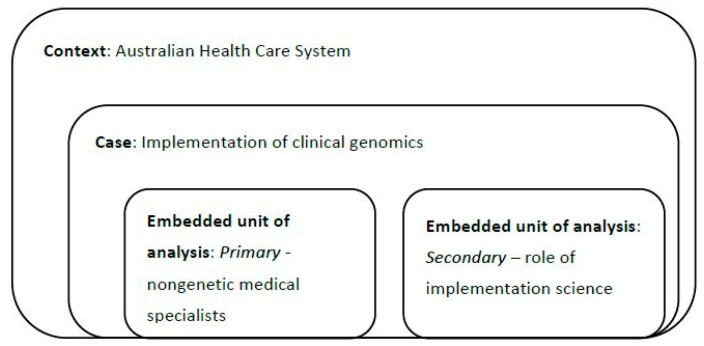
Single case, multiple embedded design [14].

We used a single case, multiple embedded case study design adopting a multi-level mixed methods approach (Figure 1) [14]. Here, the single case is factors relating to the implementation of clinical genomics, in the context of the Australian health care system, with the use of implementation science and the experiences of nongenetic medical specialists embedded within the case.

Clarity of assumptions made is an essential step when using case study methodology [14]. We have focused on the experiences of nongenetic medical specialists, rather than genetic clinicians, as we considered these individuals to be central to successful implementation. Their experiences will enable us to identify gaps in what is known and consider how we can address them. 

### 2.3. Participants and Recruitment

Nongenetic medical specialists who were working in the field of genomics on either an Australian Genomics or Melbourne Genomics hybrid effectiveness/implementation project were eligible to take part in the study. The project teams included clinical geneticists, medical specialists (e.g., neurologists, oncologists, nephrologists, immunologists etc.) laboratory scientists and commonly a genetic counsellor. Potential participants were identified by an expert reference group that consisted of five people with genetic/genomic, clinical, laboratory and implementation science knowledge and experience [18]. Everyone identified was invited to interview via email (SB).

### 2.4. Data Collection, Procedure and Analysis

#### 2.4.1. Interview Development

Formative, non-theory informed or designed research exploring the barriers to adoption of clinical genomics has revealed frequently identified challenges to implementation, for example, environment and resources. To enable a deeper interrogation of the influences on implementation of clinical genomics, we used the Theoretical Domains Framework (TDF) to shape the interview schedule [18] to ensure we explored topics and ideas that are more difficult to articulate without structured prompts, such as emotion and incentives [19]. A less well considered schedule e.g., “tell me about any barriers to implementation” is unlikely to provide rich data on e.g., social influences, while “tell me about the people who have influenced how you work in genomics” may reap more informative data. The TDF is an evidence-based behaviour change framework comprising of 14 domains and can provide a detailed understanding of the influences on behaviour. An essential first step is the identification of the “target behaviour” [20], i.e., the behaviour to be addressed. For many interventions this can be straightforward, however, exploring the complexity of the implementation of clinical genomics required the development of target behaviour areas (TBAs). These areas spanned the patient journey from TBA 1: ensuring appropriate patients are selected for genomic testing, TBA 2: requesting testing and interpreting the data and TBA 3: providing results to patients.

#### 2.4.2. Conducting the Interview

Participants provided verbal consent at interview following review of the participant information sheet. Given the complex nature of implementing clinical genomics and the time constraints of clinicians, we were keen to maximise our interview time. As a result, we developed an outline process map for the use of clinical genomics with key activity points or challenges, grouped by TBA, with prompts for the key areas for exploration. This tool ensured clinicians were not surprised by any of the questions, could remain focused on the relevant phase of the patient journey under consideration and amend processes that were incorrect. One experienced qualitative researcher (SB) undertook all the interviews, face to face. The interview schedule can be found in Supplementary Table S1. The interviews typically lasted 60 min, were audio-recorded, fully transcribed, de-identified and managed in NVivo 11. Transcripts were given an identifier code NGMS 1, 2, 3, etc.

#### 2.4.3. Data Analysis

Analysis was led by the use of the TDF to gain a comprehensive understanding of the barriers to achieving each TBA. However, the TDF can be challenging for people without behaviour change expertise to engage with and we were keen to maximise the dissemination of our findings. Therefore, we used the Capability, Opportunity and Motivation Behaviour (COM-B) framework [21] alongside the TDF to double code barriers. The COM-B is a theoretically informed model of behaviour change, aligned with the TDF, with three domains enabling categorisation of behavioural barriers according to “capability”, “opportunity”, or “motivation”. The coding of the COM-B and TDF are related, for example, TDF code belief about consequences influences motivation and therefore aligns with the COM-B code of motivation. The definitions of TDF and COM-B codes along with the association between the two frameworks can be found in the Supplementary Files (Supplementary Tables S2 and S3). Dual coding provided the advantage of the in-depth analysis of the TDF, which is useful for future strategy development and simplicity for communication using the COM-B.

To begin with, transcripts were examined for barriers and intuitively identified enablers (i.e., enablers we think might work but do not have a theoretical underpinning) by TBA to identify factors that facilitate or hinder the implementation of genomics in clinical practice. Since participants were involved in providing genomic testing in clinical care within either Australian Genomics or Melbourne Genomics clinical research project barriers and enablers specific only to research were removed to draw out “real life” challenges and facilitators. The decision to remove the research barriers was agreed by the expert reference group and undertaken by SB, along with JL and NT. Each barrier was considered and discussed in relation to the broader genomic context and the current research related infrastructure. For those barriers identified as research-related, we then discussed how these research-related barriers would play out in the real world and the extent to which they would affect implementation. If it was very little/not at all, then they were considered research-related barriers. Common barriers from the remaining data were grouped and analysed deductively using the COM-B and TDF. Intuitive enablers, identified by interviewees, were noted alongside the “real life” grouped barriers (i.e., barriers participants had experienced in practice) providing key priority areas to inform future intervention strategy design. Initially, five transcripts were coded independently by two researchers (SB and JL) and compared for discrepancies. One researcher (SB) completed the coding with ongoing regular meetings (JL and NT) to discuss and resolve challenging coding and findings. Finally, recurring themes with their associated TDF coding were identified (SB, JL and NT).

## 3. Results

First, we present the demographics of the participants who participated in the study and then the TDF and COM-B findings by TBA. 

### 3.1. Demographics

Participants represented a range of medical specialities (Table 1). Most participants (*n* = 6) worked in Nephrology, followed by neurologists (*n* = 4). All the participants, bar one from Queensland, were from Victoria.

### 3.2. Target Behaviour Areas 1–3

Priority areas were identified across the clinical pathway outlined in Table 2, Table 3 and Table 4. For each TBA, all the grouped barriers identified are listed and, using just one of these barriers as an example, an exemplar quote is provided. The associated TDF and COM-B code is noted, with reasoning, and an example of an intuitive enabler with an exemplar quote. Additional detail of the barriers and intuitive enablers with exemplar quotes for each TBA can be found in the Supplementary Tables (Supplementary Table S4).

### 3.3. Target Behaviour Area 1

In the early stages of using genomic testing TBA 1, ensuring appropriate patients receive testing, we found seven barriers (see Table 2): gaining belief about genomics; gaining confidence (i) in themselves and (ii) trust in others; setting the tone for the environment; gaining genetic skills; the process of “doing genomics”; developing genomic clinical skills; managing the evolution of knowledge and skills. Considering just one of these barriers, “gaining belief about genomics”, participants here did not appreciate the full value of doing the test because they were too busy and felt engaging in genomics took too long, “Well, you’d have to ask others why they haven’t had a lot of buy in... we’ve tried to include others [clinicians] but, we have busy clinics here”, NGMS11. This barrier is TDF coded as *Belief About Consequences* and COM-B code *Motivation* as clinicians did not see or appreciate the value of genomic testing. Participants reported seeing results from genomic testing made a difference and a suggested intuitive enabler as, “the results from the project—people like me need to be out there presenting it at hospital fora and things saying this is changing the way we’re doing things”, NGMS6.

### 3.4. Target Behaviour Area 2

For TBA 2, test selection and interpretation, we identified three priority areas (with four sub-themes) (Table 3): the need for role clarity; the need for preparation; corporate knowledge which had four sub-themes of still learning, safety in being vulnerable, trust between professionals and building relationships. Taking just one of these barriers, “developing corporate knowledge: safety in feeling vulnerable” during test selection and interpretation nongenetic medical specialists are not experts in this field, “Q: How did you feel when you first came out of the labs, more to the bedside. A: Yeah. I was petrified.” NGMS9. This barrier is TDF coded as *Emotion*, and COM-B code *Motivation*, as clinicians felt uncomfortable not understanding the process. Participants suggest an intuitive enabler as getting involved and building relationships “(I am) much more comfortable now having been involved in the clinic and I think for some of my fellow colleagues Um, for some of them, I think, you know, it’s (clinical genomics) completely off the radar as well” NGMS12.

### 3.5. Target Behaviour Area 3

Finally, during TBA 3 communicating results to patients, there were two barriers (Table 4): comfort in communicating genomic results to patients and managing bureaucracy. When examining the barrier “comfort in feeding results back to patients”, participants report the challenges that arise from working in isolation in the evolving field of genomics, “This is new, most people don’t have their head around it”, NGMS11. This barrier is coded as TDF code *Professional Identity*, and COM-B code *Opportunity*, because clinicians are still working out their professional place in clinical genomics. Several intuitive enablers were identified to overcome this barrier including formal feedback from genetics professionals, “I think having a more formal feedback (from genetic professionals) is certainly appropriate and to be honest mostly from colleagues and having contact with the geneticists and, genetic counsellors. And having that regular contact, where I wouldn’t necessarily if ever, sort of met them prior to this”, NGMS12.

## 4. Discussion

This case study had two embedded units of analysis in relation to the factors affecting the uptake of the implementation of genomics: the perceptions of nongenetic medical specialists and the role of implementation science. We consider each area before discussing the approach that will be taken to develop targeted interventions to support novice nongenetic medical specialists.

### 4.1. Primary Embedded Unit of Analysis: Nongenetic Medical Specialists

When considering the findings by TBA, the initial step of TBA 1, ensuring appropriate patients are offered genomic testing, presented the most barriers. This result suggests TBA 1 is the most challenging step for nongenetic medical specialists and that supporting practitioners through this period will be essential to ensure successful implementation. Some of the barriers identified are well established e.g., gaining genetic knowledge [22] and skills [23] though other barriers are less well evidenced e.g., gaining belief about genomics (understanding the value of doing the test) and gaining trust in others. During TBA2, test ordering and selection, three barriers (the need for role clarity, developing corporate knowledge and the need for preparation) also included several subthemes including challenging topics to reveal e.g., safety in feeling vulnerable, trusting other professionals and acknowledging that they were still learning. These subthemes may be transient barriers and are important to capture and act on for future novice nongenetic medical specialists as they start engaging with clinical genomics. Relationships with and levels of reliance on clinical geneticists will also evolve as the nongenetic medical specialists overcome the barriers to implementation and grow in their autonomy in their use of genomic testing. While the later stage of TBA 3, communicating results to patients, identified relatively few barriers (comfort in communicating results to patients and managing bureaucracy), this final TBA was perceived as less challenging and will require fewer interventions to support new nongenetic medical specialists looking to apply clinical genomics in their everyday practice. Genomic information is acknowledged to come with some uncertainty [24] and—perhaps surprisingly—when communicating results to patients, our participants often reported this step as just part of what clinicians do, “That’s a call I make”, NGMS6. While it is essential that the nongenetic medical specialists were comfortable in communicating results, this TBA was reported as a more process-orientated step.

### 4.2. Secondary Embedded Unit of Analysis: Role of Implementation Science

The field of implementation science has much to offer those interested in seeing clinical genomics successfully integrated into practice, from a health systems perspective to framing the focus of clinical studies to include implementation into practice or for the systematic examination of the processes of implementation. In this study, we centre on the use of two behavioural frameworks to provide an in-depth investigation into factors influencing the uptake of clinical genomics by nongenetic medical specialists. Designing and assessing the impact of an intervention to overcome these barriers, however, may benefit from the use of additional implementation methods (e.g., behaviours change techniques [25], or ERIC strategies [26]) and frameworks (e.g., Proctor’s Implementation Outcomes Framework [27]) [28].

Careful structuring of the research process with implementation science concepts has maximised the depth of the findings generated from this study and demonstrates the potential to leverage clinicians’ experiences with theory-informed interventions. In a complex field, such as the implementation of clinical genomics, narrowing down the area of interest, by using the TDF TBAs prior to interview, enables specificity while allowing spillover across different clinical disciplines. Using the TDF domains to develop our interview schedule allowed focus to be placed on challenging areas that non-behaviour change theory-informed interviews sometimes do not probe or do not uncover [19]. Our study revealed barriers to implementation that go beyond practitioners’ surface-level concerns to generate more considered responses, for example, TDF code *intentions* (i.e., clinicians’ making a conscious decision to consider using genomic testing) and *emotion* (i.e., clinicians personal feelings related to the use of genomics in their clinical practice).

Previous studies have identified *Environmental Context and Resources, Beliefs about Consequences, and Knowledge* as central to clinician decision making for genetic testing [29]. Our study, however, provides a more nuanced view, demonstrating how barriers change over the patient journey which can be seen at first glance through the COM-B findings (Table 2, Table 3 and Table 4). Examining the findings in more detail with the TDF shows some domains were present throughout the whole clinical journey i.e., *professional identity, social influences* and, *environmental context and resources*. The prevalence of these three TDF domains suggests these are priority areas [30] to focus on for facilitating nongenetic medical specialists to successfully adopt clinical genomics. Ensuring clinical genomics fits with nongenetic medical specialists’ perceptions of their own professional identity, interpersonal interactions between professionals and that resources are sufficient will be important regardless of the stage of the clinical journey. These detailed data will be of value when designing theory-based targeted interventions [31] to support nongenetic medical specialists implement clinical genomics into their practice.

### 4.3. Theory-Based Targeted Interventions to Support the Implementation of Clinical Genomics

The use of behavioural theory in practice is challenging but an essential step [32] to developing targeted interventions to support novice nongenetic medical specialists apply clinical genomics in practice. A theory-based approach, with the use of TBAs and TDF coding, ensures findings can be generalised [5] to comparable contexts and permits the identification of potential evidence-based interventions that may promote the implementation of clinical genomics for nongenetic medical specialists.

It is possible to anticipate some of the interventions required to support clinicians [33]. For example, during TBA 1, three barriers (the process of “doing” genomics, developing genomic (clinical) skills and gaining genetic knowledge) i.e., TDF codes *knowledge* and *skills* may logically respond to an education intervention [21]. However, other barriers are less clear cut and using the TDF allows for systematic identification of theory-informed behaviour change techniques [34] alongside participants’ intuitive enablers [35]. The COM-B, while informative, does not narrow down attention and so behaviour change strategies may not reach the underlying cause of a presenting barrier. In contrast, the TDF permits detailed analysis using the live TDF intervention evidence base (https://theoryandtechniquetool.humanbehaviourchange.org/tool (accessed on 23 February 2021)). For example, in TBA 1 the TDF code of *belief about consequences* from the barrier of gaining belief about genomics should respond to the behaviour change technique of “salience of consequences”. In context, this may present as providing evidence of the impact of genomics, for example through seeing a patient receive a diagnosis from genomic testing) (see examples in Table 5). From this study, we are now ideally placed to identify interventions to supplement the intuitive enablers identified by participants at interview (Supplementary Table S4) to support the adoption of clinical genomics.

**Figure 2:**
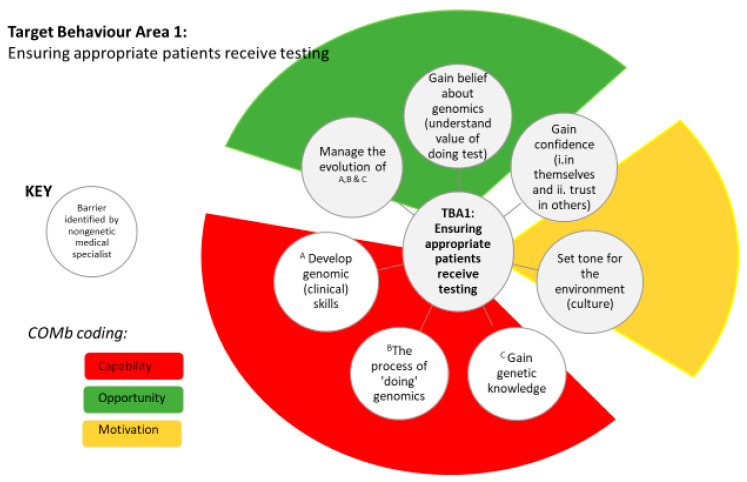
Target behaviour area 1 barriers: demonstrating ease communication through coding by the Capability, Opportunity and Motivation Behaviour framework.

Dual coding barriers with the TDF and COM-B provides a theory-informed behaviour change lens that maximises this study’s findings. Firstly, through the COM-B the results are easily accessible to clinicians and policy makers in the field. Figure 2 demonstrates how the simplicity of the COM-B can be clearly articulated in graphic form. This immediate impact on dissemination is essential to ensure engagement and a tendency towards theory-informed interventions to support nongenetic medical specialists adopt clinical genomics. Secondly, coding with the TDF allows for more explicit identification of appropriate evidence-supported interventions. Attention to this step is essential to prevent the current waste in research where learning from research is stifled [36]; http://www.thelancet.com/campaigns/efficiency/statement (accessed on 23 February 2021).

### 4.4. Limitations

This study has limitations. Clinical genomics is still in its infancy and so the participants in this study are relatively early adopters. The challenges they have faced will be common to others though there may be other barriers, not revealed here, associated with clinicians who have not yet taken up clinical genomics. This study was undertaken within one health system. All countries have their own idiosyncrasies and so generalising findings internationally will need a considered approach. However, through the use of implementation science theory, we have a strong starting point with standardised categories that have context-specific barriers and interventions that could be explored in other settings or countries. Adopting this approach will help reduce the need for others to reinvent the wheel and also contribute to advancing the science of implementation. We interviewed 16 nongenetic medical specialists and achieved data saturation with the latter interviews yielding less new data. Our sample included a large proportion of nephrologists (*n* = 6) which may reflect the active nephrology community’s uptake of clinical genomics.

## 5. Conclusions

Our findings promote a considered approach to theory and behaviour change framework selection to identify priority areas to support nongenetic medical specialists and inform future implementation strategies to promote the adoption of clinical genomics. To maximise the value created by health care research for patients and consumers, it is imperative that we continue to support the development of the quality of implementation evidence generated by clinicians and researchers. Without the use of theory to understand nongenetic medical specialists’ challenges when implementing clinical genomics, risks the development of misaligned implementation interventions that do not meet nongenetic medical specialists needs. Gaining a theory-informed insight into the barriers and enablers nongenetic medical specialists encounter when implementing clinical genomics is essential to guide the identification of appropriate practical interventions to support the take up of clinical genomics in practice.

## Figures and Tables

**Table 1:** Participant demographics by clinical speciality.

Clinical Speciality	No. of Participants
Neurology	4
Cardiology	1
Nephrology	6
Immunology	2
Oncology	3
**Subtotal**	16

**Table 2:** Target behaviour area 1 (ensuring appropriate patients receive testing) grouped barriers coded by the Theoretical Domains Framework and Capability, Opportunity and Motivation (COM) Behaviour framework. Detailed information about the barriers and enablers reported for each grouped barrier can be found in the Supplementary Files (Supplementary Table S4).

COM	Barriers	Example Quote (Related to the Bolded Barrier)	Enablers
**C**	Gain belief about genomics e.g., understanding the value of doing the test (TDF domain: belief about consequences)		
	There is a lack of natural referral patterns, and time requirements, which make it more challenging to appreciate the value of testing as clinicians are time poor	We have busy clinics here. This, you know, one of the things that (this genomics project is) supposed to be done in the context of a routine clinic. No chance. No chance at all. NGMS11	Seeing results NGMS 1, 2, 6
	Gain confidence in (i) themselves (ii) trust in others e.g., to gain/grow i.e., genetic knowledge and skills (TDF domain: i) belief about capabilities and ii) social influences)		
**O**	Challenging to gain/grow genetic knowledge and skills because they are unable to join meetings to grow confidence, not trained to counsel, lack of experience, lack of GC at offering stage to build confidence	I learnt a huge amount from our genetic counsellors on how they consent. I don’t think, to start off with, I would have appreciated that that was important and I think, because my experience would be fairly reflective of most (physicians), nobody told me that I needed to worry so much about incidental findings ‘cause nobody—no other (physicians) appreciated that so I didn’t know that so I didn’t do that or I wouldn’t have done it. NGMS4	Having knowledgeable person to aske informal questions NGMS2 CGs and GCs to support NGMS3 Lab experience NGMS5 Gaining experience NGMS9 Informal discussions with colleagues NGMS15 Research programme experience NGMS1 Easy to explain to patients in research setting NGMS13 Access to other physicians with genetic knowledge NGMS12
	Set the tone for environment (culture) e.g., running meetings (TDF domain: social influences)		
	How meetings are run	No barriers noted—enabler coded	Congenial relationships NGMS16 Personal contact with physicians NGMS3 Small local meetings best for info sharing NGMS4
**M**	^A^ Gain genetic knowledge e.g., Who to refer? What conditions might have a genetic basis? (TDF domain: knowledge)		
	Need to know who to refer and what conditions might have a genetic basis. There is a gap in understanding of who to select, lack of awareness of how many conditions may be genetic and a need for people to understand the whole process not just the test	The issue is that people (nongenetic medical specialists) really need to change thinking because people (nongenetic medical specialists) don’t think about this being genetic. NGMS7	Dynamic and fluid checklists for selecting patients NGMS13 Interim gatekeeping as knowledge grows NGMS13 Training NGSM12
	^B^ Find out about the process of “doing” genomics e.g., information about consent processes, how to access services (TDF domain: skills)		
	Lack of information about consent processes with no (formal) consent training and how to access services with no centralised/established path	I think historically we haven’t been particularly well trained as adult physicians (about consent). NGMS12	Clear referral criteria Having an informal checklist (clinical reasoning) NGMS10-links to confidence
	^C^ Develop genomic (clinical) skills e.g., getting hands-on (TDF domain: skills)		
	Getting hands-on with the process of clinical genomics	I think the evolution of clinical expertise and practice over years (helps gain clinical skills). Did I go to any training course to talk about it or anything like that? No. It’s just something that you, another bit of information and a skill that you acquire. NGMS10	Seeking feedback, reflecting NGMS4 Gained during the research process NGMS7 Scientists “dumbing down” information NGMS8—ties to gain confidence
**C**	Managing the evolution of motivation barriers ^A,B,C^ e.g., knowledge shifting, processes changing (TDF domain: professional identity and belief about capabilities)		
	Knowledge is shifting and the evidence base is dynamic and fluid, so processes are changing. The lack of guidance leads to relying on own clinical experience, and hindered by a lack of genomic literacy	Even if we were to limit our interest to (one clinical area) genetics, it’s still a very dynamic and fluid field. Even the most enthusiastic have a hard time to keep up with the research discoveries—which happen, almost on a monthly basis, you have a new gene associated with a condition. NGMS1	“Generational shift” younger practitioners will come in with knowledge NGMS5 Summaries of latest evidence NGMS3

Green—opportunity; Red—capability; Yellow—motivation; ^A^ Develop genomic (clinical) skills; ^B^ The process of “doing” genomics; ^C^ Gain genetic knowledge.

**Table 3:** Target behaviour area 2, (test selection and interpretation) grouped barriers coded by the Theoretical Domains Framework and Capability, Opportunity and Motivation Behaviour framework. Detailed information about the barriers and enablers reported for each grouped barrier can be found in the Supplementary Files (Supplementary Table S4).

COM	Barriers	Example Quote (Related to the Bolded Barrier)	Enablers
**C**	Role clarity e.g., different craft groups undertake different roles (TDF domain: professional identity)		
	Lack of clinician genetic literacy Risk of over-enthusiastic/lack of confidence in call variants	I didn’t really understand a lot of this—the genomics science stuff and then the scientist didn’t really understand because we were talking about the clinical phenotypes—which, I think, is actually really important. NGMS2	Understanding clinician and scientist sides NGMS2 Allowing evolution of roles as comfort grows NGMS14 Meetings to break down barriers NGMS8
**O**	Need for preparation e.g., practitioners need to research and plan in order to participate (TDF domain: environmental context and resources)		
	Lack of time and effort into learning new approach	What is challenging is the variant interpretation and that’s where, I think, your average (medical specialist) is not going to have the time or interest to invest. NGMS16	Everyone is engaged and able to speak openly NGMS4
Developing corporate knowledge: the idea that everyone is collectively developing their knowledge, commonly through MDTs and through other avenues			
**O**	Developing corporate knowledge: building relationships (TDF domain: social influences)		
	Not understanding each other’s roles No meetings	I think, it would be nice to have a particular person or go-to person, that you have a good relationship with that you can just, kind of, go bounce off questions or ideas without having to even sometimes formally… maybe, a different genetic counsellor and different, it’s just a little bit hard to know where to go. NGMS2	Communication NGMS1 Regular constructive meetings NGMS8
**M**	Developing corporate knowledge: trust between professionals (TDF domain: emotion)		
	Less trust when there are no MDTs Trust in labs	“Well what’s the turnaround time?”, “Do they trust the results from that lab?” NGMS12	Important to develop trusting relationships with genetics NGMS9 Meetings to discuss patients NGMS8 Good clinical trust permits discussion NGMS5
	Developing corporate knowledge: safety in being vulnerable (TDF domain: emotion)		
	Embarrassment at not understanding the process. Lack of comfort calling variants alone	All that kind of the jargon about how relevant a variant might be, I found that was just all a bit of gobbledygook, and I, kind of, had to stop and ask, you know, at first I was a bit embarrassed because I thought everyone else knew and then I realised that the person next to me who was a clinician also had no idea what they were talking about, so it was just, kind of, yeah, and it was just good to acknowledge at the beginning that these are two different languages and how we’re going to meet in the middle. NGMS2	Getting involved NGMS12 Access to support when needed NGMS2 Positive open culture NGMS4
	Developing corporate knowledge: still learning (TDF domain: intentions)		
	We’re still learning Still need support of other professionals	I’m still very much learning? Very much learning about what it all means and very much guided by—we’re very lucky here ‘cause we do have the geneticists and things, so they can help talk a little—“well this is what it means”. So I’m learning. NGMS12	Discussion promotes “automatic” learning, NGMS9

Green—opportunity; Yellow—motivation.

**Table 4:** Target behaviour area 3, grouped barriers coded by the Theoretical Domains Framework and Capability, Opportunity and Motivation Behaviour framework. Detailed information about the barriers and enablers reported for each grouped barrier can be found in the Supplementary Files (Supplementary Table S4).

COM	Barriers	Example Quote (Related to the Bolded Barrier)	Enablers
**O**	Managing bureaucracy (TDF domain: environment and resources)		
	Bureaucracy Vague reports Speed of results	(Challenges from the delay in getting results) Often we’ve forgotten that we’ve tested (the patient) until next time they come and goes, “Oh, yes, what happened to that genetic testing”. They (NGMS) go, “Oh, look yes, we do have a result. Um, it’s going to be too hard for me to, interpret it for you now, give me a day and I’ll talk to people and then get back to you”, type of scenario. NGMS15	Clear lab reports NGMS15 Clarity in letters to referrers NGMS4
**M**	Comfort in communicating genomic results to patients (TDF domain: professional identity and social influences)		
	Evolving field Working in isolation	Things evolve and so—and the art in what we do or the challenge in what we do is deciding at which stage to intervene. NGMS10	Professional confidence (“it’s a call I make”) NGMS6 Access to experts NGMS3 Proximity to experts NGMS9 Relationships with genetic professionals NGMS12 Genomics experience NGMS5 Preparation NGMS4

Green—opportunity; Yellow—motivation.

**Table 5:** Examples of applying behaviour change theory in practice.

Barrier	TDF Code	TDF Theory Informed Behaviour Change Technique	Applied in Context
The need to gain belief about genomics, and an appreciation of the value of doing genomic testing	Belief about consequences	e.g., “salience of consequences”	e.g., providing evidence of the impact of genomics, for example through seeing a patient receive a diagnosis from genomic testing
The need to gain confidence in themselves and trust in others	Belief about capabilities	e.g., “demonstration of the behaviour”	e.g., providing an observable example, for example with a video or observing others using clinical genomics in clinic

## Data Availability

Additional data supporting this study can be found in Supplementary Table S4.

## Supplementary Materials

The following are available online at https://www.mdpi.com/2073-4425/12/2/317/s1, Table S1: Interview schedule reproduced from Taylor et al., 2018 [18] Creative Commons Attribution Non-Commercial (CC BY-NC 4.0) license. Table S2: COM-B definitions in context [37]. Table S3: TDF definitions in context [38]. Table S4: Detailed information on barriers and enablers reported by target behaviour area for each barrier.
