# Supplementary material for: Investigating the Adoption of Clinical Genomics in Australia. An Implementation Science Case Study
Source: Genes (Basel). 2021 Feb 23;12(2):317. doi: 10.3390/genes12020317 (PMC7926693; doi:10.3390/genes12020317)
Supplement: Supplementary file 1 [file genes-12-00317-s001.zip › Supplementary tables S1_2_3_ R1.docx]

**Supplementary files**

**Table S1.** Semi Structured Interview schedule reproduced from Taylor et al., [18] Creative Commons Attribution Non Commercial (CC BY-NC 4.0) license. Could you tell me what your role is and how you have been involved with the use of genomics in the clinical setting?

| **Ensuring appropriate patients receive exome sequencing**  **(Emotion,** Reinforcement, Behavioural regulation) | **TDF Domain** |
| --- | --- |
| What measures are in place to assure yourself that you are selecting the appropriate patients? | Behavioural regulation |
| Is there anything in place that makes this process work well routinely? | Reinforcement |
| What were your experiences of starting off offering testing - how did you become more proficient? What helped (or would have helped)?  Have you always been comfortable offering genomic testing in your clinical practice? What changed your mind? | Skills, Beliefs about capabilities, Beliefs about consequences,  Emotion |
| What are your experiences of managing expectation?  How have you found this? What has made it easier? | Exploratory q from patient consultation Emotion |
| **Test ordering and interpreting variants**  **(Emotion,** Reinforcement) | **Domain** |
| What do you see as your role in determining the pathogenicity of a variant and its clinical significance)? (Who’s role is it, how do you feed in?)  What would you (or did you) need to participate in the curation process (if you feel this is part of your role)? | Skills and knowledge |
| There is a large focus on multidisciplinary variant interpretation meetings as a way of interpreting results. What works/doesn’t work?  What interventions have been put in place to aid the way they work?  How would you find variant interpretation if these didn't exist? | Reinforcement  Emotion |
| Final decision making around variant classification – is there a standard process in place? | Reinforcement |
| **Providing results to patients. (Communicating results)**  **(**Emotion, Goals, Optimism**, Reinforcement, Behavioural regulation)** | **Domain** |
| Have there been (are you aware of) any issues around communicating results back to patients?  *No*: What has been put in place to ensure it worked well?  *Yes*: What has been challenging? What needs to be put in place to overcome this? | Exploratory |
| Has a routine process been established?  How do you manage this? | MAD, Behavioural regulation |
| When results are uncertain how do you feel about feeding this back to the patient?  How would you support a less experienced doctor with this?  What gives you confidence that this process is being handled well? | Emotion    Optimism |
| Has a routine process been put in place for reanalysis of results?  How do you feel about this? | Reinforcement  Emotion |
| **Incorporation into practice**  **(**Emotion, **Reinforcement)** | **Domain** |
| What do you feel should be put in place (if anything) to incorporate genomic testing into standard clinical practice?  How could this be facilitated? (is there anything other than funding) that is needed? How do we support people to change (attitudes, behaviours, habits, skills)? | Reinforcement |
| Are you happy to play a role in mainstreaming genomics? | Emotion |
| What sort of role do you envisage? | Prof ID |
| How do you keep up with the evolving evidence base? | Skills, knowledge |
| Is your organisation supportive of adopting genomics in clinical practice? In what way? | Organisational Knowledge |
| Is there anybody (or any role) outside your organisation who is key to ensuring mainstreaming? | Prof role, environmental context |
| For others starting out now, what advice would you give – maybe what you did or wish you had known? |  |

Are there any other barriers, maybe one you have overcome, that we haven’t discussed and you would like to share?

**Table S2.** COM.B definitions in context. Definitions in context were developed using existing literature [29] and reviewed by the expert reference group.

| **COM** | **Sub division** | **Definition**  [37] | **Definition in context**  ***Barrier*** | **Definition in context**  ***Enabler*** | **Associated behaviour change interventions** [21] |
| --- | --- | --- | --- | --- | --- |
| **Capability** | Psychological and Physical | Knowledge, skills and abilities to engage in the behaviour | **A lack of** knowledge, skills or ability (either organisational or individual) to engage in the implementation of genomics in the clinical setting | Knowledge, skills or ability (either organisational or individual) **to engage** in the implementation of genomics in the clinical setting | Education  Training  Enablement |
| **Opportunity** | Physical and Social | Outside factors which make the behaviour possible | Outside factors **that hinder (or do not encourage)** an organisation or individual to implement genomics in the clinical setting | Outside factors **that enable** an organisation or individual from the implementation of genomics in the clinical setting | Restriction  Environmental restructuring  Enablement |
| **Motivation** | Automatic and Reflective | Brain processes which direct our decisions and behaviours…  that energize and direct behaviour, (…not just goals and conscious decision-making also inc habitual processes, emotional responding, analytical decision-making) | Brain processes that energise or direct decisions or behaviour **away from (or not towards)** implementing genomics in the clinical setting | Brain processes that energise or direct decisions or behaviour **towards** implementing genomics in the clinical setting | Education  Persuasion  Incentivisation  Coercion  Environmental restructuring  Modelling  Enablement |

**Table S3.** TDF definitions in context **with associated COM.B codes.** Definitions in context were developed using existing literature [29] and reviewed by the expert reference group.

| **COM-B codes** | **TDF domain** | **TDF domain definition** [38] | **Definition in context** |
| --- | --- | --- | --- |
| Capability  (Psych & Physical) | Knowledge | An awareness of the existence of something | Clinicians’ actual awareness and understanding (through education/training) of the principles and process of using genomic testing in clinical practice |
|  | Skills | An ability or proficiency acquired though practice | Clinicians’ actual physical and psychological ability or proficiency acquired through actual practice (as opposed to education/training – cannot get skills through education) to make decisions whether or not to use genomic testing in practice |
|  | Memory, Attention and Decision Processes | The ability to retain information focus selectively on aspects of the environment and choose between two or more alternatives | Clinicians’ ability to remember to consider clinical genomics alongside other interventions for health risk identification, diagnosis, management, and therapy |
|  | Behavioural Regulation | Anything aimed at managing or changing objectively observed or measured actions | Clinicians’ self-created or self-imposed regulation to help make decisions about using genomic testing |
| Opportunity (Social & Physical) | Social Influences | Those interpersonal processes that can cause individuals to change their thoughts, feelings, or behaviours | Interpersonal interactions between professionals that can influence clinicians’ thoughts, feelings or behaviours (ie anything in Motivation) regarding the use of genomic testing |
|  | Environmental Context and Resources | Any circumstance of a person’s situation or environment that discourages or encourages the development of skills and abilities independence, social competence and adaptive behaviour | Any external circumstance of a clinicians’ situation or environment that clinicians consider discourages or encourages them to use genomics in practice, including impacting the development of capability, motivation or social opportunity to using genomics |
| Motivation  (Automatic & reflective) | Social/Professional Role and Identity | A coherent set of behaviours and displayed personal qualities of an individual in a social or work setting | Clinicians’ perceived professional role and identity in relation to the use of clinical genomics |
|  | Beliefs about Capabilities | Acceptance of the truth, reality or validity about at ability, talent, or facility that a person can put to constructive use | Clinicians’ perception about their own capability to consider genomic testing (terms used in literature: confidence, comfort, control) |
|  | Optimism | The confidence that things will happen for the best or that desired goals will be attained | Clinicians’ optimism or pessimism around the use of genomics in clinical practice |
|  | Beliefs about Consequences | Acceptance of the truth, reality, or validity about outcomes of a behaviour in a given situation | Clinicians’ perceptions about the value of using genomics in clinical practice – whether it is worthwhile in that it will improve patient outcomes in their own practice (term used in literature: attitude) |
|  | Intentions | A conscious decision to perform a behaviour or a resolve to act in a certain way | Clinicians’ intentions to consider using genomic testing |
|  | Goals | Mental representations of outcomes or end states that an individual wants to achieve | Whether the use of genomic medicine is a priority within their clinical practice |
|  | Reinforcement | Increasing the probability of a response by arranging a dependent relationship, or contingency, between the response and a given stimulus | Incentives, rewards, sanctions, reinforcement at any level (eg patient satisfaction; better client health; economic incentives) that encourage or increase clinicians’ decisions to use genomics in clinical practice |
|  | Emotion | A complex reaction pattern, involving experiential, behavioural, and physiological elements, by which the individual attempts to deal with a personally significant matter | Clinicians feelings related to the use of genomics in their clinical practice |
